# A longitudinal study looking at and beyond care recipient health as a predictor of long term care home admission

**DOI:** 10.1186/s12913-017-2671-8

**Published:** 2017-11-09

**Authors:** Raquel S. D. Betini, John P. Hirdes, Donna S. Lero, Susan Cadell, Jeff Poss, George Heckman

**Affiliations:** 1 University of Waterloo, 200 University Avenue West, Waterloo, ON N2L 3G1 Canada; 2 University of Guelph, 50 Stone Rd E, Guelph, ON N1G 2W1 Canada; 3 Schlegel Research Institute for Aging, 250 Laurelwood Dr, Waterloo, ON N2J 0E2 Canada

**Keywords:** Carer, Coresidence, Distress, interRAI, Relationship

## Abstract

**Background:**

The unpaid care provided by informal caregivers allows care recipients to live longer in their homes, which often results in fewer unnecessary long term care home (LTCH) admissions. Although the relationship between care recipient’s health characteristics and institutionalization is well known, the influence of caregiver distress and caregiving coresidence and relationship on this outcome is less clear. This study examines the association of care recipient care needs, caregiver distress and caregiving coresidence and relationship with care recipient long term care home admission.

**Methods:**

A total of 94,957 resident assessment instruments-home care (RAI-HC), completed between April 01st 2013 and April 01st, 2014 as part of a clinical practice by 14 Local Health Integration Networks (LHINs) in Ontario, Canada, were linked to LTCH admissions within 1 year after completion of the first RAI-HC assessment. Cox models were used to examine whether care recipient health care needs, caregiver distress and caregiving characteristics such as coresidence and relationship were associated with LTCH admission. Age, marital status and gender of the care recipient were included as covariates in the model.

**Results:**

Care recipient health care needs and age were the strongest predictors of LTCH admission followed by caregiver distress and caregiving coresidence and relationship. Care recipient marital status was not significant in the survival model. Interestingly, care recipients who were cared for by a coresiding adult child caregiver were less likely to be admitted to a LTCH than care recipients cared for by a spouse caregiver coresiding or not with care recipient. Hazard rates (HR) of admission for care recipients cared for by caregivers coresiding and with other type of relationship with care recipient were not significantly different than HR of care recipients cared for by coresiding child caregivers.

**Conclusions:**

These results emphasize the influence of caregiver distress in LTCH admission and highlight the impact of caregiving relationship and coresidence on this outcome. Policy and decision makers should consider these findings when developing and evaluating interventions aiming to avoid LTCH admissions. Moreover, caregiving coresidence and relationship should be explored in future studies with similar aims, as this information has been neglected in past research.

## Background

The unpaid care provided by informal caregivers represents a substantial economic contribution to the health care system, especially considering the high cost of institutionalization into a long-term care home (LTCH) [1–3]. Caring for older individuals with high care needs often results in considerable burden on informal caregivers [4] that can increase distress or the perception that caring demands exceed their resources [5]. These feelings of distress affect caregiver’s quality of life and their ability to continue caring activities, that eventually, leads to the institutionalization of the care recipient [6–8].

Care recipient health characteristics that demand extensive assistance or vigilance from a caregiver such as behavioral symptoms, activities of daily living (ADL) and cognitive impairment have been consistently associated with caregiver distress and institutionalization [9–12]. The latter association could be due to the presence of care recipient care needs that may not be easily manageable in a home care setting regardless of the caregiver’s willingness to provide care, when an LTCH admission becomes the best option for providing appropriate care [6, 13, 14]. Thus, it is paramount to untangle caregiver distress and care recipient health care needs in studies on predictors of LTCH admission by including these factors in multivariable models.

However, caregiver distress and care recipient health characteristics are not the only factors associated with a LTCH admission. Features related to the caregiving relationship with care recipient such as coresidence status and type of relationship (e.g., spouse, child), may also play a role in the institutionalization of the care recipient. For example, several studies reported that spouse caregivers are less likely to institutionalize their partners than non-spouse caregivers [15, 16] and they seem to have different reasons for institutionalization compared to caregivers with other type of relationship with care recipients such as adult child caregivers [17].

Because spouse caregivers are more likely to live with the care recipient than caregivers with other types of relationship, it is possible that coresidence influences caregiving experiences and ability to continue in the role since caregiver poor quality of life has been associated with coresidence [18–20]. However, there is also evidence that caregivers that do not coreside with their care recipients may be less committed to provide care than those coresiding. This has been particularly reported among older adults opting for having a stable relationship without living together (‘living apart together-LAT’) [21, 22]. With an aging population the number of older adults living this type of relationship is expected to increase [23] while its impact on institutionalization remains unknown.

Caregiving coresidence and relationship and its association with LTCH admission have not been discussed together in previous studies. Indeed, most research looking at predictors of LTCH have failed to include both caregiving related characteristics. Additionally, few studies have examined these characteristics together with caregiver distress and care recipient health care needs as predictors of LTCH admission [24, 25] or have used large datasets combined with advanced statistical models for this aim. This type of information is crucial for developing customized strategies to assist informal caregivers in their role and reduce avoidable care recipient institutionalization.

Thus, this study aims to perform an exploratory analysis by incorporating these variables (caregiving coresidence and type of relationship, caregiver distress and care recipient health characteristics) in a multilevel model to evaluate their likelihood of predicting LTCH admission among home care clients in Ontario. A variable combining caregiving coresidence and relationship (spouse, child, other) will be included in the model to better understand how these variables act together as predictors to LTCH admissions. It is expected that this variable will be significantly associated with institutionalization with an unknown direction. Moreover, it is expected that care recipients with high care needs and those cared for by distressed caregivers will be more likely to be admitted to LTCH.

## Methods

### Context

Home and community care (HCC) agencies funded by 14 Local Health Integration Networks (LHINs) in Ontario assess the care needs of individuals living at home. Case managers or care coordinators working at these agencies develop customized care plans often involving a wide range of health-care professionals (e.g., nurses, physiotherapists, social workers, registered dieticians and personal support workers). Long-stay clients (> 60 days receiving care) typically receive their initial assessments (i.e., RAI-HC) during the first visit of the case manager.

This longitudinal observational study included the most recent clinical assessments of long-stay home care clients in Ontario to evaluate care recipient and caregiver characteristics associated with LTCH admissions.

### Sample

To test the hypothesis of this longitudinal study, a total of 94,957 long-stay home care clients’ RAI-HC assessments were included in the analysis. They were completed between April 01st 2013 and April 01st, 2014 as part of a clinical practice by 14 LHINs (which constitutes the total number in the whole province). The most recent RAI-HC assessment occurring during the sample period was included in this analysis. Only assessments of individuals identified as residing in ‘Private home/apartment – with or without home care services’ were included in the dataset as other types of living arrangements such as assisted care living or group homes are not in the focus of this study. Assessments conducted in the hospital setting were excluded.

Demographic and health related information of long stay home care recipients admitted and not admitted to LTCH are presented in Table 1. Nurses or social workers working as case managers in the HCCs completed the majority of the assessments. The assessments were sent to the University of Waterloo by the Health Shared Services Ontario (HSSO) through a license agreement between these two organizations. To guarantee the anonymity of the data the dataset does not include any identifier at the individual level. Information on LTCH admissions was obtained from the Client Health and Related Information System (CHRIS), a web-based care recipient management system that collects information on home care clients’ admission and discharge. This dataset was sent to University of Waterloo by HSSO through the same process and agreements used for sharing the RAI-HC assessments.

**Table 1:** Characteristics of home care clients: not admitted versus admitted to long term care home (LTCH)

*Informal caregiver characteristics*		No admission (*n* = 89,119)	LTCH admission (*n* = 5838)	*P* value
		% (*n*)	% (*n*)	
Coresidence and relationship				
	Spouse coresiding	31.4 (28,024)	32.0 (1868)	<.0001
	Spouse non-coresiding	0.64 (573)	0.77 (45)	
	Child coresiding	19.2 (17,149)	20.1 (1173)	
	Child non-coresiding	29.8 (26,569)	34.8 (2031)	
	Others coresiding	6.38 (5687)	3.31 (193)	
	Others non-coresiding	12.0 (11,117)	9.04 (528)	
Distress	Present	28.3 (25,207)	49.2 (2872)	<.0001
*Care recipient characteristics*				
Marital status	Married	39.7 (35,371)	41.6 (2432)	<.0001
Gender	Female	65.9 (58,783)	64.4 (3759)	0.0142
Age	<40	2.86 (2552)	0.22 (13)	<.0001
	40–64	14.1 (12,531)	3.94 (230)	
	65–74	15.5 (13,821)	10.5 (612)	
	75–84	32.5 (28,971)	36.5 (2133)	
	85–94	31.7 (28,316)	43.5 (2542)	
	95+	3.28 (2927)	5.28 (308)	
MAPLe^a^	Low/Mild	18.5 (16,467)	2.62 (153)	<.0001
	Moderate	39.1 (34,823)	25.4 (1486)	
	High	31.7 (28,260)	42.4 (2474)	
	Very high	10.7 (9569)	29.5 (1725)	
CPS^b^	Intact or borderline intact	50.8 (45,251)	15.6 (913)	<.0001
	Mild/Moderate	43.4 (38,730)	71.2 (4158)	
	Moderate/Severe	4.50 (4010)	12.4 (726)	
	Very severe	1.27 (1128)	0.70 (41)	
ADL-H^c^	Independent	51.0 (45,473)	31.8 (1858)	<.0001
	Supervision required/Limited impairment	31.4 (27,992)	45.1 (2635)	
	Extensive assistance required	13.0 (11,586)	19.5 (1141)	
	Dependent/total dependence	4.56 (4068)	3.49 (204)	
Dementia	Present	18.0 (16,080)	51.7 (3021)	<.0001

^a^ *MAPLe* Method of Assigning Priority Levels, ^b^ *CPS* Cognitive Performance Scale, ^c^ *ADL-H* Activity of daily living Hierarchy. Note: In some cases, the number of cases is less than the full sample size because of missing data

### Scales

#### Resident assessment instrument – home care (RAI-HC)

The RAI-HC is a standardized comprehensive tool used to assess the strengths, needs and preferences of home care clients living in the community.

Some of the assessed domains are related to function, health, social support and service use. This instrument has been widely used in North America (Canada and multiple states in the U.S.), Europe (Italy, Switzerland, Finland, Estonia, etc.), and Asia/Pacific Rim (Hong Kong, Singapore, Japan, Australia, New Zealand). The RAI-HC is part of a family of assessment systems developed by interRAI, a research collaborative from about 35 countries [26] dedicated to the improvement of quality and continuity of care across the health care sectors [27]. The RAI-HC has been extensively validated [28–30] with several studies providing evidence on the good validity and reliability of items and scales derived from the RAI-HC [30–32]. Some of the RAI-HC scales and items that were used to describe care recipient clinical health and caregiver distress are presented below.

#### The Method for Assigning Priority Levels (MAPLe)

The Method for Assigning Priority Levels (MAPLe), an algorithm related to care recipient heath care needs, has been found to be predictive of both home care clients’ LTCH admission as well as caregiver distress [11, 14, 33, 34]. This algorithm, which is derived from RAI-HC, was developed to inform health care provider decisions about the urgency of need for care among home care clients and the allocation of home care resources. The scores range from 1 to 5 with higher scores indicating a higher priority for care.

The MAPLe algorithm was derived from a study sample including close to 5000 clients from Ontario [33]. Dependent variables chosen as indirect indicators of ‘need’ for additional services and further derivation of MAPLe were: presence of signs of caregiver distress, rating oneself or being rated by others as being better off elsewhere and nursing home admissions. The final algorithm was validated using RAI-HC data from 7 countries and across Canadian provinces and include items related to: falls, behavioral symptoms, cognitive, activities of daily living (ADL) impairment and others. Further details on the derivation of MAPLe are explained elsewhere [33]. The relationship between the MAPLe scores and caregiver distress is consistent with other studies showing care recipients’ health needs involving behavioral symptoms, ADL and cognitive impairment associated with caregiver distress [9–11].

#### Activities of daily living hierarchy scale (ADL-H)

The ADL Self-Performance Hierarchy Scale reflects the disablement process by grouping ADL performance levels into discrete stages of loss. The scale measures physical functioning by assessing the individual’s capacity to perform activities related to: eating, personal hygiene, dressing, and movement (transfer and locomotion). Validation of this scale has been previously reported [29, 30, 35].

#### Cognitive performance scale (CPS)

The CPS is a hierarchical index used to rate a person’s cognitive status. The scale includes items on short and long-term memory, ability to make decisions, communication skills, and functional performance and it has been validated against the Mini-Mental State Exam (MMSE) for the detection of cognitive impairment [29, 36–39].

#### Caregiver distress variable

This variable derives from two items in the RAI-HC that correspond to assessor judgement of the following statements: 1) ‘a caregiver is unable to continue in caring activities – e.g. decline in the health of the caregiver makes it difficult to continue’ or 2) ‘primary caregiver expresses feelings of distress, anger, or depression’. The caregiver distress indicator was jointly developed by interRAI and the Canadian Institute for Health Information and it has been used to report caregiver distress by different studies and organizations [40]. Evidence from past studies has shown that this variable is associated with care recipient health symptoms that have been linked to caregiver distress [11, 14, 33, 34, 41].

### Statistical analysis

In Ontario, a total of 14 LHINs provide home care services for the community. Although LHINs share similar provincial guidelines for the provision of care, they develop their own initiatives that may affect the amount of respite provided for caregivers or the care provided for their clients. In this context, the influence of caregiver distress on LTCH admission may be different for each LHIN as well as the relationship between caregiver distress and MAPLe scores. Thus, an exploratory analysis was performed to evaluate whether the association between caregiver distress and MAPLe scores vary among LHINs (*n* = 97,493).

**Fig. 1:**
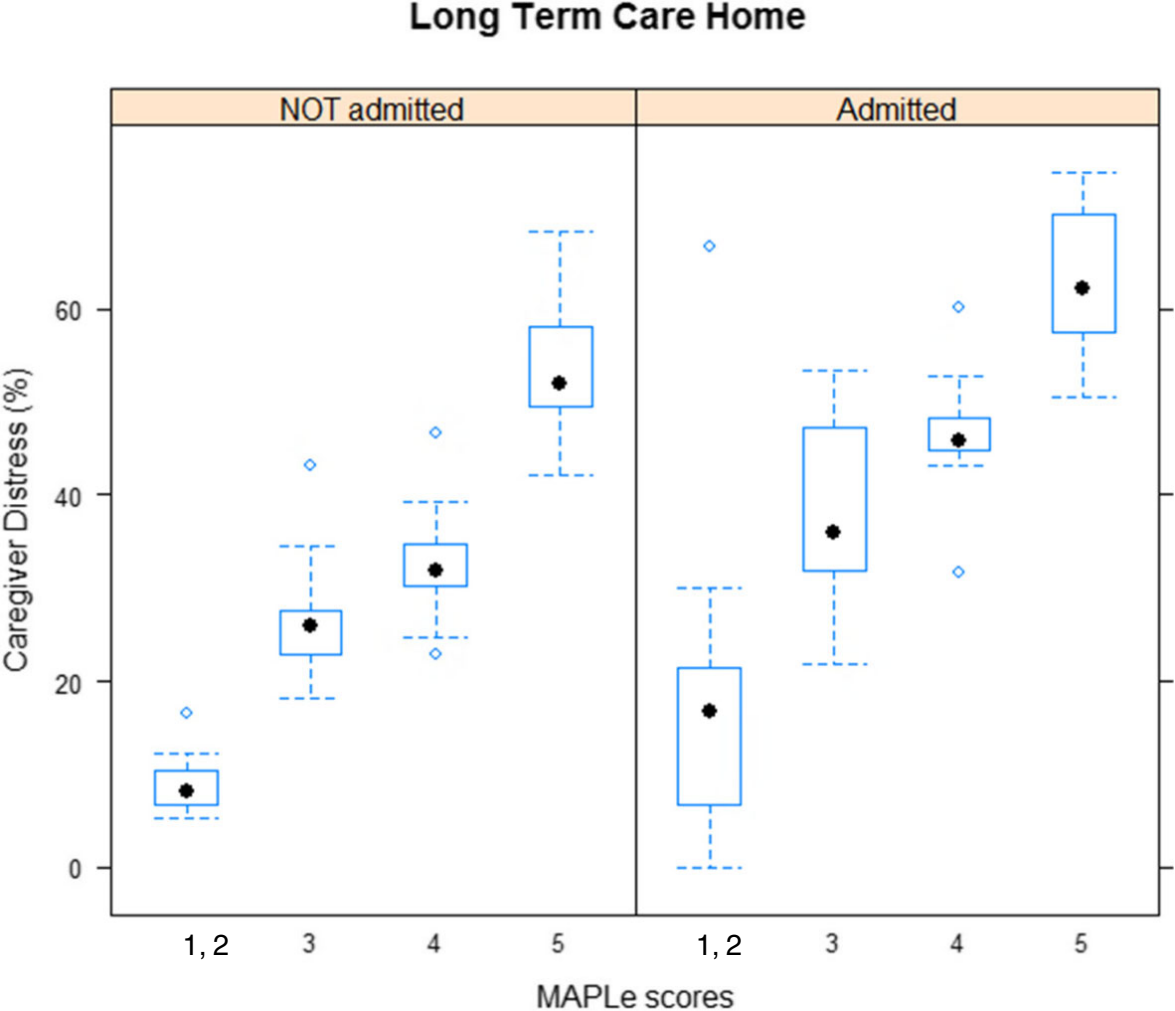
Distribution of percentage of caregiver distress by MAPLe scores and LTCH admission. The horizontal dotted line at the bottom of each plot is the sample minimum, excluding outliers; the lower limit of the box in each figure defines the lower quartile (25th percentile); the sample mean is represented by the heavy dot inside each box; the upper quartile (75th percentile), is defined by the upper limit of each box; the sample maximum, excluding outliers, is defined by the horizontal line at the top of each plot and empty circles represent the outliers

Figure 1 shows the distribution of the average proportions of caregiver distress by MAPLe scores for 14 LHINs. The results indicate that although the relationship between these variables is consistent across LHINs, the average proportion of caregiver distress by MAPLe varies among them. Also, proportions of distressed caregivers by MAPLe are higher for care recipients admitted to LTCH.

These preliminary results indicated that the following analysis should consider variations in the relationship between caregiver distress, MAPLe scores and LTCH admissions among LHINs.

### Statistical models

As a first step, a frequency analysis (chi square tests, *p* < 0.05) of RAI-HC items was performed to confirm whether the variables MAPLe, caregiver distress and caregiving variable were associated with LTCH admission and to examine whether there were other RAI-HC items that could be related to the response variable. Another study identified the items in the RAI-HC representing care recipient health characteristics associated with LTCH admission to become part of the MAPLe algorithm, which is strongly associated with institutionalization [33, 42]. This explains why no additional variables/items representing care recipient health characteristics associated to LTCH admission were identified.

Next, analysis of unadjusted models were performed to evaluate the strength of association between the independent variables identified in the frequency analysis and the dependent variable (LTCH admission) in the model. In addition, care recipient demographic information (age, gender and marital status) were also analysed in the unadjusted models as these variables were previously related to LTCH admission [43–45]. Moreover, based on preliminary analysis, a variable with 6 categories combining caregiving coresidence (yes/no) and type of relationship (spouse/child/other relationship) was created to facilitate the interpretation of the results.

Significant variables (*P* < 0.05) in the unadjusted models were included in the regression analysis of survival data based on the Cox proportional hazards models to identify the variables related to the hazard rates (HR) of LTCH admission. The regression parameters in the adjusted Cox model were estimated by using a model based covariance matrix estimate to account for the intracluster dependence [46]. The results of model-based covariance matrix estimates have been reported instead of the robust sandwich estimates because of the small number of clusters (i.e., 14 LHINs) [47]. The empirical model estimates may not be appropriate when the number of clusters is lower than 40 [47]. Clients who were not admitted to a LTCH within 365 days of the initial assessment were right-censored, of the meaning that this information was still used to estimate model parameters even though these clients did not experience the event. Survival was measured as time in days starting from the RAI-HC assessment date until date of the event. A test of proportionality was performed to examine the main assumptions of the Cox proportional hazard model. Unadjusted variables were tested in the regression model before being included in the final Cox proportional hazard model for analysis. All statistical analyses were carried out using SAS version 9.4.

## Results

### Survival analysis

All variables tested in the unadjusted models (Table 2) were significant. Except for ‘marital status’, all the significant unadjusted variables (gender, age group, MAPLe, caregiver distress, coresidence by relationship) were significant in the final model (Table 3).

**Table 2:** Survival models for unadjusted variables associated with long term care home admission

Parameters	Estimate	SE	Hazard Ratio (HR)	95% HR Confidence limits	Chi-square	Pr > Chisq
*Care recipient characteristics*						
Gender (ref. ‘female’)	0.13	0.03	1.14	(1.08–1.20)	22.1	<.0001
Married (ref. ‘no’)	0.11	0.03	1.12	(1.06–1.18)	17.6	<.0001
Age group (ref. ‘18–39 yrs’)						
40–64 yrs.	1.38	0.20	3.98	(2.27–6.96)	23.4	<.0001
65–74 yrs.	2.25	0.20	9.56	(5.52–16.56)	64.9	<.0001
75–84 yrs.	2.74	0.19	15.4	(8.94–26.60)	96.7	<.0001
85–94 yrs.	2.91	0.21	18.4	(10.68–31.76)	109.8	<.0001
95+ yrs.	3.08	0.21	21.9	(12.61–38.26)	119.1	<.0001
MAPLe (ref. ‘1’)						
2	0.36	0.16	1.44	(1.04–1.99)	4.79	0.0286
3	1.63	0.13	5.12	(3.94–6.64)	151.3	<.0001
4	2.36	0.13	10.6	(8.22–13.77)	322.3	<.0001
5	3.06	0.13	21.4	(16.54–27.78)	536.1	<.0001
*Informal caregiver characteristics*						
Caregiver distress (ref. ‘no’)						
Yes	0.88	0.03	2.41	(2.28–2.54)	1027.5	<.0001
*Caregiving features*						
Coresidence (cores) and relationship type (ref. ‘child caregiver coreside’)						
Child does not cores	0.10	0.03	1.11	(1.03–1.19)	8.61	0.0033
Spouse cores	0.01	0.04	1.01	(0.94–1.09)	0.12	0.72
Spouse does not cores	0.18	0.15	1.20	(0.89–1.16)	1.43	0.23
Others coreside	−0.72	0.07	0.48	(0.42–0.56)	86.8	<.0001
Others does not cores	−0.33	0.05	0.71	(0.64–0.79)	40.9	<.0001

**Table 3:** Multivariate survival model for long term care home admission

Parameters	Estimate	SE	Hazard Ratio (HR)	95% HR Confidence limits	Chi-square	Pr > Chisq
*Care recipient characteristics*						
Gender (ref. ‘female’)	0.06	0.03	1.07	(1.01–1.13)	4.81	0.03
Married (ref. ‘Yes’)	0.08	0.04	1.08	(0.99–1.18)	3.36	0.06
Age group (ref. ‘18–39 yrs’)						
40–64 yrs.	1.73	0.28	5.67	(3.22–10.00)	36.0	<.0001
65–74 yrs.	2.55	0.28	12.7	(7.28–22.38)	79.1	<.0001
75–84 yrs.	2.94	0.28	19.1	(10.93–33.35)	107.3	<.0001
85–94 yrs.	3.12	0.28	22.7	(13.08–39.75)	120.5	<.0001
95+ yrs.	3.28	0.29	26.5	(15.06–46.83)	128.4	<.0001
MAPLe (ref. ‘1’)						
2	0.23	0.16	1.25	(0.90–1.74)	1.91	0.16
3	1.45	0.13	4.27	(3.29–5.55)	119.1	<.0001
4	2.14	0.13	8.48	(6.55–10.99)	261.4	<.0001
5	2.71	0.13	15.0	(11.58–19.54)	412.7	<.0001
*Informal caregiver characteristics*						
Caregiver distress (ref. ‘no’)						
Yes	0.59	0.03	1.81	(1.71–1.91)	446.7	<.0001
*Other informal caregiver characteristics*						
Coresidence (cores) and relationship type (ref. ‘child caregiver coreside’)						
Child does not cores	0.34	0.04	1.40	(1.30–1.50)	82.5	<.0001
Spouse coreside	0.23	0.05	1.25	(1.13–1.39)	19.1	<.0001
Spouse does not cores	0.36	0.15	1.43	(1.06–1.94)	5.40	0.02
Others coreside	0.10	0.08	1.11	(0.94–1.29)	1.59	0.21
Others does not cores	0.33	0.05	1.39	(1.25–1.54)	37.1	<.0001

The highest HR of LTCH admission was observed for the care recipients with the highest care needs (MAPLe 5). Increased care recipient age was also related to an increased HR to be admitted to LTCH. Care recipients cared for by distressed caregivers showed higher chances to be institutionalized than those cared for by non-distressed caregivers.

Care recipients cared for by child caregivers coresiding had lower HR of institutionalization.

The HR of institutionalization of care recipients cared for by caregivers with other relationship and coresiding was not statistically different than the HR for those cared for by child caregivers coresiding. The HR of care recipient institutionalization was higher for those cared for by a spouse caregiver, regardless of living arrangement, and also for those cared for by caregivers with other type of relationship and non-coresiding.

## Discussion

Several studies have reported the influence of care recipient health characteristics on institutionalization [13, 33, 48] while others have examined the impact of caregiver related characteristics on LTCH admission [6, 49, 50]. This study brings together information on care recipient health, caregiver and caregiving characteristics and their association with institutionalization. The relationship between MAPLe scores and caregiver distress found in the preliminary analysis echoes previous reports on the same association [11, 14, 33]. This finding is in accordance with other studies in which caregiver distress related symptoms were associated with care recipient ADL impairment and behavioral symptoms [41, 51–58]. These care recipient health characteristics are part of the MAPLe algorithm, which explains the association between this scale and caregiver distress.

Of the care recipients who were admitted to LTCH, the higher proportion of distressed caregivers, as identified by MAPLe scores (Fig. 1), suggests that caregiver distress plays a role in institutionalization. Thus, caregiver distress, MAPLe scores and caregiving variables were entered in the survival models having LTCH admission as an outcome. Higher care recipient MAPLe scores were associated with higher likelihood of institutionalization. This result aligns with evidence that certain care recipient health characteristics such as psychotic symptoms and behaviour dysregulation, included in the MAPLe algorithm, are predictors of institutionalization [6, 13]. The association between MAPLe scores and LTCH admission also could be related to the presence of health care needs that surpass caregiver ability to provide care, creating a situation in which institutionalization becomes the most appropriate destination for the care recipient [52, 54, 57].

Caregiver distress was also a predictor of LTCH admission after considering care recipient health, a finding that aligns with other studies [7, 8, 59]. Research has shown that caregiver’s perception of their ability to provide care and feelings of burden are key factors contributing to their decision to institutionalize [60–63]; these factors may not be related to the actual frequency or intensity of care provided (i.e., objective burden) [64].

Care recipient age was a significant predictor of LTCH admission in the survival model. This is similar to other studies and is likely related to changes in health care needs associated with aging [65, 66]. On the other hand, the impact of caregiver coresidence with care recipient on institutionalization has been less examined.

One of the most interesting findings in the present study was the combined impact of caregiver relationship and coresidence on the likelihood of LTCH admission. More specifically, care recipients of coresiding child caregivers were less likely to be admitted to a LTCH than care recipients of spouse caregivers with the same living arrangement, non-coresiding child caregivers or caregivers with other types of relationship that do not coreside. These findings demonstrate the influence of caregiving coresidence and relationship on LTCH admission, a neglected subject in past studies. One study on this subject reported that care recipients of non-coresiding caregivers were also more likely to be admitted to a nursing home [8].

It is not clear how the coresidence between caregiver and their care recipients influence caregiver responsibilities and their ability to provide care. Some studies have reported that caregivers who live with their care recipients tend to provide instrumental care and spend more hours overseeing care recipients’ activities, especially those with behavioral symptoms [67, 68]. Although there is evidence on the negative impact of coresidence on caregiver burden or distress [18, 19, 53], this finding has not been reported consistently [69, 70].

One of the few studies reporting the influence of coresidence on distress among different caregiving relationships has shown that child-caregivers who coreside with their parent(s) experienced less strain than child caregivers who do not coreside [69]. According to the authors, child caregivers who coreside may do so due to special bonds with their parents, as opposed to a child who is unwilling to coreside. In addition, daughter caregivers coresiding with their parents have reported that the quality of their relationship is one of the reasons why they became their parent’s primary caregiver [71]. Interesting, son caregivers also have reported that they assume this role motivated out of a sense of love and obligation [72]. Thus, caregiving may likely have a positive meaning for child caregivers who live and care for their parents, as they are less likely to institutionalize. However, information on caregivers with other type of relationship with care recipient and institutionalization is scarce. As the population is aging, more individuals that are not necessarily spouse or an adult child of the care recipient will assume the caregiver role, justifying the inclusion of this group in studies like this one.

The association between the type of caregiver relationship and care recipient institutionalization has been discussed in the past, with most studies providing evidence that non-spousal caregivers are more likely to institutionalize their family member or friend [7, 62, 73]. Again, the reason for LTCH admission seems to differ depending on the type and quality of the caregiving relationship. A recent study on reason for care recipient LTCH admission showed that spouse-caregivers reported more reasons related to themselves (e.g., burden), but child caregivers more often reported reasons related to care recipient health as motivations for LTCH placement [17]. Interestingly, Savundranayagam [74] observed that stress and relationship burden were positively associated with intention to institutionalize among spouse and child caregivers respectively.

It is also possible that the higher HR of institutionalization among care recipients cared for by coresiding spouse caregivers compared to those cared for by coresiding child caregivers is related to the older age of many spouse caregivers as caregiver-aging related [75] health issues have been associated with institutionalization [6, 59, 76]. Spousal caregivers are also more likely to be in the caregiver role for a longer time compared to child caregivers, becoming more emotionally and physically vulnerable [77]. In contrast, the higher likelihood of institutionalization among care recipients of spouse caregivers who do not coreside could be related to other reasons. Spouses who are living apart sometimes are not fully committed to provide care for their partners [21, 22]. For example, many women opt for this type of relationship to avoid the unequal demands of caring that she has experienced in a previous marriage [78].

This study has limitations that should be acknowledged. The items on caregiver distress and ability to continue are dichotomous variables which provide only a yes/no type of answer. Thus, it is not possible to distinguish levels of distress that could have yielded a stronger association with LTCH admission. In addition, the RAI-HC focus is on the care recipient. Therefore, other caregiver information that may be related to a caregiver’s reasons to institutionalize was not collected and therefore could not be included in the analysis. It should also be acknowledged that policy and other contextual factors that may affect LTCH placement were not considered in this study.

One of the major strengths of this study is the inclusion of a large number of assessments completed as part of a routine practice that encompasses care recipient health characteristics, caregiver distress, and caregiving information allowing for a more detailed examination of the potential predictors of LTCH admission.

## Conclusions

This study demonstrates that care recipient health needs is an important predictor of LTCH admission as well as caregiver distress and caregiving characteristics, such as relationship and coresidence with care recipient. The later information has been neglected and should be considered in future studies investigating predictors of LTCH admission and also when developing interventions to reduce institutionalization. Caregiver distress can be prevented by respite while caregiving experiences can be improved by education and support services that ultimately allow them to continue in their role without compromising their quality of life. In contrast, interventions that target care recipients with high care needs may not be always effective as often care recipient health decline is unavoidable and institutionalization becomes the most suitable option.

## Abbreviations

ADL: Activity of daily living
ADL-H: Activity of daily living Hierarchy
CIHI: Canadian Institute for Health Information
CPS: cognitive performance scale
GEE: Generalized estimating equations
HCC: Home and community care
HR: Hazard ratio
HSSO: Health shared services Ontario
IADL: Instrumental activity of daily living
LHIN: Local health integration network
LTCH: long term care home
MAPLe: Method of assigning priority levels
SAS: Statistical analysis system
